# Editorial: Case reports in pediatric urology 2022

**DOI:** 10.3389/fped.2023.1161074

**Published:** 2023-03-15

**Authors:** Walid Farhat

**Affiliations:** Department of Urology, School of Medicine and Public Health, University of Wisconsin, Madison, WI, United States

**Keywords:** congenital urological anomalies, pediatric urolithiasis, ureteropelvic junction (UPJ) obstruction, xanthogranulomatous pyelonephritis, giant bladder diverticulum, situs inversus totalis (SIT), unicystic, yolk sac tumor

**Editorial on the Research Topic** Case reports in pediatric urology 2022

The ability to prenatally diagnose urogenital anomalies allows clinicians to tailor treatment and further refine the management of the pathology at hand. The first case report describes a 2-year-old girl diagnosed with pediatric urolithiasis at 20 weeks gestation (Bose et al.). Postnatally, the stone was extracted and a ureteropelvic junction obstruction was found and repaired. The child had a recurrent stone one and a half years later. Prenatal kidney stone diagnosis is rare but can occur in some cases. While there is limited literature available on this topic, a few studies have been conducted to explore the diagnosis and management of prenatal kidney stones. Prenatal diagnosis of kidney stones could be challenging, as the stones may not be visible on standard ultrasound scans. However, advanced imaging techniques, such as fetal magnetic resonance imaging (MRI), could be used to detect and diagnose kidney stones prenatally. While prenatal kidney stone diagnosis is rare, it can have serious consequences if not properly managed.

Xanthogranulomatous pyelonephritis (XGPN) is a rare, chronic, and destructive form of kidney infection that is characterized by the accumulation of lipid-laden macrophages, chronic inflammation, and tissue destruction. It most commonly affects middle-aged women, but can also occur in men and children. The diagnosis of XGPN in children can be challenging, as it may present with nonspecific symptoms such as fever, abdominal pain, and urinary tract symptoms. Imaging studies such as ultrasound, computed tomography (CT), or magnetic resonance imaging (MRI) may be helpful in the diagnosis of XGPN, but biopsy or surgical resection is often necessary to confirm the diagnosis. The second case report describes a rare case of Xanthogranulomatous pyelonephritis (XGPN) in a 7-year-old boy infected with Staphylococcus aureus (Deng et al.). The child underwent partial nephrectomy and the conclusion highlights the importance of early diagnosis and treatment of XGPN to reduce its morbidity and mortality. XGPN is an aggressive pathology that frequently requires total nephrectomy. The authors were able to salvage the remaining kidney and provide a precedent for other surgeons to consider.

Giant bladder diverticulum (GBD) is a rare condition that can result in complications such as urinary tract infections, stone formation, and obstructive uropathy. Situs inversus totalis (SIT) is another rare condition in which the position of the internal organs is mirrored from their usual positions. The diagnosis of GBD in a patient with SIT can be challenging, as it may present with nonspecific symptoms such as dysuria, frequency, urgency, and suprapubic pain. Imaging studies such as computed tomography (CT) or magnetic resonance imaging (MRI) may be helpful in the diagnosis of GBD, but cystoscopy is often necessary to confirm the diagnosis. The management of GBD in a patient with SIT typically involves surgical intervention to remove the diverticulum, but the approach may vary depending on the size and location of the diverticulum. In some cases, an open surgical approach may be necessary, but laparoscopic bladder diverticulectomy has been shown to be a safe and effective alternative.

The third case report describes a 14-year-old male patient with a combination of giant bladder diverticulum and situs inversus totalis (SIT), who underwent successful laparoscopic bladder diverticulectomy (Chen et al.). The report highlights the technical points and difficulties of performing the surgery in such cases and the importance of understanding the rare combination of bladder diverticulum and SIT in children. Authors are commended for using minimally invasive approaches to tackle lower urinary tract pathology such as diverticulum. The proximity of the diverticulum to the ureters or vas in a male patient could be challenging but applying minimal access with a panoramic view could be the way to address these rare pathologies.

UCPDN is a rare variant of Wilms tumor characterized by a cystic component and partially differentiated cells that affect children. It is characterized by the presence of a cystic component and partially differentiated cells. It typically presents in young children and is diagnosed through a combination of clinical and radiological findings, as well as histopathological examination of the tumor tissue. Treatment involves surgical resection of the tumor, and the prognosis is generally good. The fourth case report describes the first case of unicystic cystic partially differentiated nephroblastoma in a child (Chao et al.). The patient underwent laparoscopic decortication, radical nephrectomy, and postoperative chemotherapy. The report highlights the challenge in the diagnosis, differential diagnosis, and treatment of unicystic renal tumors in pediatric cases. Though prognosis for UCPDN is generally good, with a 5-year survival rate of 90% to 100%. However, the prognosis may depend on the stage and grade of the tumor, as well as the extent of surgical resection.

Testicular YST commonly presents as a painless scrotal mass or swelling in children and adolescents. However, the clinical presentation of YST can be nonspecific, and the diagnosis may be delayed or missed. The diagnosis is based on a combination of clinical, radiological, and histopathological findings, and misdiagnosis can occur and delay the appropriate management of the patient. Treatment involves surgical resection of the tumor, and the prognosis is generally favorable. The treatment of testicular YST involves surgical resection of the tumor. Chemotherapy and radiation therapy may be considered in cases of advanced or metastatic disease, but their role in the treatment of testicular YST is not well-established. Overall, the prognosis for testicular YST is favorable, with a 5-year survival rate of 90%–95%. The last case report describes a 2-year-old pediatric patient with a giant testicular yolk sac tumor that was misdiagnosed as orchitis by color Doppler ultrasonography (Wang et al.). The case highlights the importance of measuring α-fetoprotein levels in the initial diagnosis of testicular yolk sac tumors to aid in accuracy. Suspicious testicular masses in children should be thoroughly evaluated with tumor markers as well as a low threshold for surgical interventions.

We thank the authors for sharing their experiences and reviewers for improving the quality of these manuscripts.

